# Indigenous–non-Indigenous inequalities in oral health prevention among children and adolescents in Ecuador: a cross-sectional study

**DOI:** 10.1186/s12939-026-02928-6

**Published:** 2026-06-24

**Authors:** Edy Quizhpe, Miguel San Sebastián

**Affiliations:** 1 Centro de Investigación en Salud Pública y Epidemiología Clínica (CISPEC), Facultad de Ciencias de la Salud “Eugenio Espejo”, Universidad UTE, Quito, Ecuador; 2 Hospital General del Sur de Quito, IESS, Quito, Ecuador; 3 Department of Epidemiology and Global Health, Umeå University, Umeå, Sweden; 4 Instituto de Epidemiología y Salud Comunitaria “Manuel Amunárriz”, Hospital Franklin Tello, Nuevo Rocafuerte, Ecuador

**Keywords:** Inequalities, Ecuador, Oral health, Children, Ethnicity

## Abstract

**Background:**

Although oral diseases are among the most prevalent chronic conditions affecting children and adolescents globally, access to preventive and treatment services remains uneven. These disparities are particularly pronounced among Indigenous populations and reflect long-standing structural and social inequities. This study examined the prevalence of, and ethnic inequalities in, refraining from dental care and inadequate toothbrushing frequency among Indigenous and non-Indigenous children and adolescents in Ecuador.

**Methods:**

A cross-sectional study was conducted using a nationally representative data from the ENSANUT 2018 demographic survey. Two preventive oral health outcomes were included: refraining from dental care in the previous 12 months and inadequate toothbrushing frequency (< 2 times/day). Ethnicity was self-reported and categorized as Indigenous or non-Indigenous (Afro-Ecuadorian, Mestizo, White, and Montubio). Survey weighted prevalence and absolute risk differences between ethnic groups were estimated to assess inequalities in the outcomes, with adjustment for sex, place of residence and region, and stratified by age group.

**Results:**

Refraining from dental care was more prevalent among Indigenous than non-Indigenous children (50.6% vs. 37.3%), corresponding to an adjusted absolute risk difference of 9.2% points (95% CI: 4.4 – 13.9). Inadequate toothbrushing was consistently higher among Indigenous children and adolescents, with an adjusted absolute difference of 12.8; 95% CI: (9.8 – 15.9) and 11; 95% CI: (7.8 – 14.3) percentage points, respectively.

**Conclusions:**

Substantial ethnic inequities in access to dental care services and preventive oral health behaviour persist among children and adolescents in Ecuador. These findings underscore the need for equity-oriented and culturally appropriate oral health policies targeting Indigenous populations.

## Introduction

Oral diseases remain a significant global public health concern, affecting approximately half of the world’s population, with a disproportionate impact on children and adolescents [1]. At present, dental caries, periodontal diseases, and untreated oral disorders are considered chronic conditions resulting in pain, prolonged infections, compromised nutritional status, reduced academic performance, and a deterioration in quality of life. Despite their prevalence, oral diseases can be largely preventable through cost-effective interventions, such as timely use of dental care services and routine toothbrushing with fluoridated toothpaste [2, 3].

The World Health Organization (WHO) and the World Dental Federation recommend routine visits to dental health professionals as a key oral health prevention strategy. Likewise, brushing your teeth at least twice daily with fluoridated toothpaste is fundamental to preventing oral disease and a key component of public health strategies [4]. Nevertheless, disparities in the use of these preventive practices and access to oral services persist, often reflecting pronounced social and ethnic inequalities [2, 5].

Ethnic inequities in oral health are commonly seen around the world, particularly within Indigenous communities [5–7]. A U.S. report found that 70% of Navajo children had untreated dental caries, indicating higher rates of dental problems among Indigenous children compared to non-Indigenous (16%) [8]. Similarly, a systematic review found that Indigenous children in Australia had twice the rate of dental caries than other groups (European and Asian Australians) [9]. These disparities commonly stem from long-standing structural inequalities such as poverty, remote living conditions, a lack of culturally appropriate services, and experiences of discrimination [10, 11]. Access to oral health prevention initiatives is often unequal from early life, with disadvantages accumulating over time and contributing to persistent oral health problems across the life course, from childhood and adolescence into adulthood [12, 13]. These challenges occur alongside an epidemiological transition among Indigenous peoples, moving from infectious diseases to chronic non-communicable diseases. This shift is further shaped and intensified by long-standing social determinants of health [14, 15].

These patterns are also reflected in Ecuador, where oral health and access to preventive oral health services remain uneven. A study in 2018 revealed that nearly two-fifths (39.2%) of children and adolescents experienced dental problems that affected their quality of life during the previous 12 months. Significant disparities were also observed, particularly among children residing in rural areas and Indigenous populations [16]. Another study from 2012 reported that approximately 30% of Ecuadorian children had not attended a dental visit in the previous 12 months [17]. Despite the Ministry of Public Health (MoPH) having established a national oral health plan in 2009 [18], dental caries, periodontal disease, and fluorosis remain significant public health concerns among young people [19, 20]. According to the official 2019 National Institute of Statistics and Census (INEC) report on children’s health, only 75% of children regularly brush their teeth according to international recommendations, and just over half had accessed oral health services in the previous year [21].

In Ecuador, Indigenous populations have historically experienced significant socioeconomic marginalization and continue to encounter structural and persistent inequalities that hinder their access to preventive dental care, despite nationwide efforts to expand universal health coverage (UHC) [22, 23]. Oral health research focusing specifically on Indigenous children and adolescent remains scarce, highlighting an important knowledge gap. Nevertheless, available evidence points to substantial disparities in oral health outcomes. One study investigating the relationship between oral status and food insecurity reported a higher prevalence of oral health problems among Indigenous children (39.5%) compared with their mestizo peers (19%) [24].

Similarly, disparities have been observed in oral health-related quality of life, with Indigenous children and adolescents reporting a greater negative impact from dental conditions (43.6%) than mestizos (38.6%) [17]. Socioeconomic and behavioural factors further compound these inequities. A study using a national representative sample found that clinical examinations of children in Indigenous communities revealed a dental caries prevalence of 77%, of which 87% of lesions remained untreated. These outcomes were associated with lower maternal education levels and high consumption of sugar-containing products [25].

No previous studies have focused on the potential ethnic inequalities in oral preventive measures among young people in the country. Therefore, this study aimed to examine the prevalence of, and ethnic inequalities in, refraining from dental care and inadequate toothbrushing frequency among Indigenous and non-Indigenous children and adolescents in Ecuador.

## Methods

### Study design

A cross-sectional design, using a nationally representative data from the 2018 national survey of health and nutrition (Encuesta Nacional de Salud y Nutrición - ENSANUT in Spanish) carried out by –the INEC [26], was applied. The ENSANUT is a demographic and health survey designed to assess the population’s health and nutritional status, covering areas such as chronic diseases, dietary habits, physical activity, and health care access. Data were collected through direct interviews with either the head of the household (mother or father) or a qualified informant (anyone over 12 years old competent of providing accurate information); adolescents (from age 12) completed the questionnaire themselves. The survey includes two components: a household section and an individual section, each comprising several standardized questionnaires designed to collect information relevant to the survey objectives. The present analysis uses data from the specific oral health practices questionnaire [27]. A total of 23,621 participants were included in this study, consisting of 13,700 children (55.50%) and 9,891 adolescents (44.50%). The age groups were defined according to the Economic Commission for Latin America and the Caribbean (Comisión Económica para América Latina y el Caribe - CEPAL in Spanish) classification, whereby children were categorized as individuals aged 5–11 years and adolescents as those aged 12–18 years [28].

### Variables

Two indicators reflecting access to prevention and promotion were selected: refraining from dental care and inadequate toothbrushing frequency (ITF). Refraining is commonly used to monitor health system performance and to assess progress towards UHC. Refraining is defined as avoiding seeking health care despite needing it, and it is used to assess oral health service coverage [29]. This information was obtained through the survey question: “In the past 12 months, have you visited the dentist?” Respondents who answered “no” were asked for “What was the main reason for not visiting the dentist?” and those that indicated one or more reasons such as a lack of nearby dental or health care facilities, high costs, insufficient funds, limited time, or other barriers to accessing dental care services were classified as children and adolescents who refrained from seeking dental services. Self-reported tooth-brushing frequency, meanwhile, can serve as a proxy measure for oral hygiene [29, 30]. ITF was captured by asking participants: “How frequently do you brush your teeth?” response options included once a week, two or three times a week, once a day, two or more times a day, or never. Toothbrushing was considered inadequate if participants reported brushing their teeth fewer than two times per day [2, 31].

Ethnicity was determined based on the self-identification of the head or another informed respondent. Children and adolescents were classified as Indigenous or non-Indigenous (Afro-Ecuadorian, Mestizo, White, and Montubio). Sex/Gender (male, female), place of residence (urban, rural), and geographical region (Andean, Coast, Amazon) were included as covariates, given their well-established role as social determinants of oral health and their potential influence on access to preventive dental care.

### Statistical analysis

The population characteristics were summarized using descriptive statistics to calculate the prevalence of the two outcomes stratified by age group and ethnicity. Regression analysis was applied to estimate absolute risk differences (ARD) between ethnic groups and the outcomes using the non-Indigenous group as the reference. First, an univariable model (model 1) was estimated, followed by a multivariable model (model 2) adjusted for sex, place of residence and region. Statistical inference was based on 95% confidence intervals (CI). Sample weights were applied to all analyses which were performed using the Stata version 17.1 statistical software.

## Results

### Population characteristics

Table 1 shows the socioeconomic characteristics of the study population. No age-group differences were observed in the Indigenous population regarding sex; however, most children and adolescents lived in rural areas (71.3% and 73.6%), in the Andean (58.0% and 65.4%), and the Amazon regions (31.1% and 27.2%). Conversely, non-Indigenous children and adolescents lived in urban areas (70.9% and 68.0%), in the Coast (56.0% and 54.5%) and Andean regions (39.6% and 40.9%).

**Table 1:** Demographic and geographic characteristics of ecuadorian children and adolescents in 2018 (weighted samples)

	Indigenous (*n* = 3260) (%)			No Indigenous (*n* = 20361) (%)			
	Children	Adolescents	Total	Children	Adolescents	Total	
**Sex/Gender**							
Men	927 (52.63)	740 (49.44)	1667 (51.16)	5768 (50.91)	4636 (51.34)	10,405 (51.10)	
Women	834 (47.37)	757 (50.56)	1592 (48.84)	5561 (49.09)	4394 (48.66)	9955 (48.90)	
**Residence**							
Urban	506 (28.77)	395 (26.40)	902 (27.68)	8043 (70.99)	6148 (68.09)	14,192 (69.70)	
Rural	1255 (71.23)	1102 (73.60)	2357 (72.32)	3286 (29.01)	2882 (31.91)	6168 (30.30)	
**Region**							
Andean	1004 (58.01)	963 (65.49)	1968 (61.45)	4322 (39.62)	3561 (40.95)	7883 (40.21)	
Coast	187 (10.82)	107 (7.30)	294 (9.20)	6109 (56.00)	4742 (54.53)	10,851 (55.35)	
Amazon	539 (31.17)	400 (27.22)	940 (29.35)	477 (4.38)	393 (4.52)	870 (4.44)	

### Socioeconomic inequalities in oral health

The prevalence of the two oral health outcomes is displayed in Table 2. Indigenous children had a higher prevalence of refraining from seeking oral health services (50.6%) than the non-Indigenous reference group (37.3%), while Indigenous adolescents had a slightly higher prevalence (46.4% vs. 40.5%). Regarding ITF, both Indigenous children (43.1%) and adolescents (31.9%) had a higher prevalence than their non-Indigenous counterparts (26.4% and 18.8%, respectively).

Table 2 also presents the absolute risk differences for refraining from seeking oral health services and for ITF. Both the crude and the adjusted models revealed statistically significant differences between the Indigenous and non-Indigenous groups for both outcomes, except for the adolescents’ group in refraining. Additionally, in the two outcomes higher differences between Indigenous children compared to Indigenous adolescents were found.

Indigenous children showed a higher statistically significant difference in refraining of 9.2% points (pp) compared to the non-Indigenous group (ARD = 9.2; 95% CI: 4.4 – 13.9) in the adjusted model. The ethnic difference in inadequate toothbrushing was even higher and statistically significant (ARD = 12.8; 95% CI: (9.8 – 15.9)).

Indigenous adolescents, like the children’s group, showed higher levels of refraining than the reference group, although to a lesser extent, (ARD = 2.4; 95% CI: (-2.3 – 7.3), and not statistically significant. In terms of ITF, similarly to the children group, Indigenous adolescents had a 111 pp statistically significant difference than the non-Indigenous (ARD = 11; 95% CI: 7.8 – 14.3).

**Table 2:** Prevalence and absolute ethnic differences in oral health outcomes among children and adolescents, Ecuador 2018

Variable		Children			Adolescents	
	Prevalence (%)	Model 1	Model 2**	Prevalence (%)	Model 1	Model 2**
**Refrain from seeking oral health services**						
** N**on-indigenous	37.3	0	0	40.5	0	0
Indigenous	50.6	13.3 (8.8 – 17.8) *	9.2 (4.4 – 13.9) *	46.4	5.9 (1.2 – 10.5)	2.4 (-2.3 – 7.3)
**Inadequate toothbrushing frequency**						
Non-Indigenous	26.4	0	0	18.8	0	0
Indigenous	43.1	16.7 (13.8 – 19.6) *	12.8 (9.8 – 15.9) *	31.9	13.1 (9.9 – 16.2) *	11.0 (7.8 – 14.3) *

*Statistically significant; ** Adjusted for sex, place of residence and region

## Discussion

The results have shown that, in 2018, Indigenous children had a higher prevalence of refraining from visiting oral health services and of ITF compared with their non-Indigenous peers. These disparities persisted among adolescents for ITF, but not for refraining.

The prevalence of refraining observed in our study was similar to that reported among Indigenous populations in countries such as Peru and Australia, where prevalences of approximately 60% and 50%, respectively, have been documented [32, 33]. A systematic review showed that factors such as certain cultural practices, poor dietary patterns and limited access to oral healthcare services were more common among Indigenous children, which may help explain the observed ethnic disparities [34, 35]. Our study found that ITF was more prevalent among Indigenous than non-Indigenous children and adolescents, with prevalence estimates of 43.1% and 31.9%, respectively, among Indigenous participants. However, these estimates were lower than those reported in previous studies of the Ecuadorian youth population (58% among children and 50% among adolescents), which included both Indigenous and non-Indigenous groups [36, 37]. While the adoption of a comprehensive and intercultural strategy by Ecuador’s Ministry of Public Health for child and adolescent health within healthcare and educational environments [38, 39] may partly explain this positive trend, dental caries continues to represent a major public health issue among infants. Recent studies continue to report high prevalence, despite the fact that dental caries is largely preventable [40–43]. A possible explanation is that public health efforts have focused more on treatment than on prevention and health promotion [44]. Moreover, the national oral health plan has not been updated since 2009, and preventive strategies, such as encouraging twice-daily brushing and implementing fluoridation initiatives, have not achieved the desired impact. This may reflect persistent social inequalities, suggesting that current approaches are not sufficiently accessible, culturally appropriate, or effective across all population groups [45].

Addressing oral health among children and adolescents requires intercultural strategies that recognize the interconnected social, cultural, and structural determinants operating across multiple levels within a complex system [46]. Programs designed with cultural considerations, particularly those grounded in strong community participation and collaboration with local health professionals and community organizations, have demonstrated effectiveness not only in Indigenous communities, but also in the broader population [47].

In addition, effective population-level interventions, such as water and salt fluoridation, remain under-implemented by local and national authorities, increasing exposure to oral health risks [48, 49]. Although improvements at the individual-level, such as increased toothbrushing frequency, may occur, structural barriers to accessing healthcare continue to contribute to adverse oral health outcomes [50]. Finally, our results indicate that Indigenous children experienced a greater burden than adolescents, suggesting, as reported in other studies, that preventive interventions should begin early in life [51].

### Methodological considerations

Several issues should be considered when interpreting the results of this study. A key strength of the study is the nationally representative sample of children and adolescents. However, as it is common in population-based surveys, exposures and outcomes were reported by the head of the household on behalf of the children, which may have introduced reporting or recall bias.

Furthermore, the adjustment strategy was restricted to the variables available in the dataset, and key socioeconomic factors such as household income, parental education, and health insurance were not included; therefore, residual confounding cannot be ruled out, and the observed associations should be interpreted with caution.

## Conclusion

This study revealed significant ethnic disparities in oral health among Indigenous children and adolescents in Ecuador. Early childhood-focused, regionally and culturally tailored school-based oral health programs are recommended, particularly for rural areas. Monitoring inequality using ethnicity, gender, and geography-specific indicators is essential to ensure the goal of UHC in Ecuador.

## Data Availability

Data used in this study are publicly available and can be retrieved from https://www.ecuadorencifras.gob.ec/documentos/web-inec/Estadisticas_Sociales/ENSANUT/ENSANUT_2018/Principales%20resultados%20ENSANUT_2018.pdf.

## **Abbreviations**

WHO: World Health Organization
MoPH: Ministry of Public Health
UHC: Universal Health Coverage
ENSANUT: Encuesta Nacional de Salud y Nutrición
INEC: Instituto Ecuatoriano de Estadísticas y Censos
CEPAL: Comisión Económica para América Latina y el Caribe
ITF: Toothbrushing Frequency
ARD: Absolute Risk Differences
CI: Confidence Intervals
